# Batch Fabrication of Microelectrode Arrays with Glassy Carbon Microelectrodes and Interconnections for Neurochemical Sensing: Promises and Challenges

**DOI:** 10.3390/mi15020277

**Published:** 2024-02-15

**Authors:** Emma-Bernadette A. Faul, Austin M. Broussard, Daniel R. Rivera, May Yoon Pwint, Bingchen Wu, Qun Cao, Davis Bailey, X. Tracy Cui, Elisa Castagnola

**Affiliations:** 1 Department of Biomedical Engineering, Louisiana Tech University, Ruston, LA 71272, USA; eaf021@email.latech.edu (E.-B.A.F.); austinbrouss@gmail.com (A.M.B.); drr029@latech.edu (D.R.R.); 2 Department of Bioengineering, University of Pittsburgh, Pittsburgh, PA 15260, USA; m.pwint@pitt.edu (M.Y.P.); biw15@pitt.edu (B.W.); qc3bs@virginia.edu (Q.C.); xic11@pitt.edu (X.T.C.); 3 Center for Neural Basis of Cognition, University of Pittsburgh, Pittsburgh, PA 15261, USA; 4 Institute for Micromanufacturing, Louisiana Tech University, Ruston, LA 15213, USA; dbailey@latech.edu; 5 McGowan Institute of Regenerative Medicine, University of Pittsburgh, Pittsburgh, PA 15219-3110, USA

**Keywords:** glassy carbon, microelectrode arrays, xenon difluoride (XeF_2_) etching, fast scan cyclic voltammetry, serotonin

## Abstract

Flexible multielectrode arrays with glassy carbon (GC) electrodes and metal interconnection (*hybrid* MEAs) have shown promising performance in multi-channel neurochemical sensing. A primary challenge faced by *hybrid* MEAs fabrication is the adhesion of the metal traces with the GC electrodes, as prolonged electrical and mechanical stimulation can lead to adhesion failure. Previous devices with GC electrodes and interconnects made of a homogeneous material (*all* GC) demonstrated exceptional electrochemical stability but required miniaturization for enhanced tissue integration and chronic electrochemical sensing. In this study, we used two different methods for the fabrication of *all* GC-MEAs on thin flexible substrates with miniaturized features. The first method, like that previously reported, involves a double pattern-transfer photolithographic process, including transfer-bonding on temporary polymeric support. The second method requires a double-etching process, which uses a 2 µm-thick low stress silicon nitride coating of the Si wafer as the bottom insulator layer for the MEAs, bypassing the pattern-transfer and demonstrating a novel technique with potential advantages. We confirmed the feasibility of the two fabrication processes by verifying the practical conductivity of 3 µm-wide 2 µm-thick GC traces, the GC microelectrode functionality, and their sensing capability for the detection of serotonin using fast scan cyclic voltammetry. Through the exchange and discussion of insights regarding the strengths and limitations of these microfabrication methods, our goal is to propel the advancement of GC-based MEAs for the next generation of neural interface devices.

## 1. Introduction

Carbon is considered the ideal material for electrochemical sensing and presents superior electrochemical stability [1,2,3,4,5]. For the last three decades, fast scan cyclic voltammetry (FSCV) at carbon fiber microelectrodes (CFEs) has been considered the gold standard for in vivo electrochemical detection of neurotransmitters, including dopamine (DA) [6,7,8], serotonin (5-HT) [1,9], and melatonin (MT) [10,11]. The sub-second temporal resolution (hundreds of milliseconds [12,13,14]) offered by FSCV is consistent with the rapid chemical fluctuations observed at neuronal synapses, facilitating real-time neurotransmitter detection. These fast scan rates can be alternated with high frequency electrophysiological recording at the same electrode [15,16].

Despite their effectiveness, CFEs are limited to a single electrode site and, even when using carbon fiber arrays, the restriction persists at one site per fiber. This limitation hinders the full potential of multiplexed analyses, which would allow simultaneous observations of electrical and chemical activities at multiple sites using a single device. Moreover, the fabrication process for CFE arrays can be intricate and time-consuming, which may impede scalability and widespread adoption [17,18,19].

On the other hand, thanks to the recent advances in material science and lithography microfabrication techniques, flexible microelectrode arrays (MEAs) with nanometer-thick metal thin-film electrodes integrated on micrometer-thick polymer substrates—such as polyimide, parylene C, and SU-8—have been developed to match the soft nature of the brain and minimize mechanical trauma and inflammation [20,21]. These flexible MEAs, along with subcellular dimension, can record electrophysiological neural activities at millisecond-scale temporal resolution and micrometer-scale spatial resolution and have been shown to seamlessly integrate with the neural tissue and improve recording longevity [22,23,24,25].

The use of carbon materials as a substitute of metal for conductive electrodes and interconnections in microfabricated flexible MEAs would enable multichannel electrochemical sensing and thus facilitate multimodal electrochemical and electrophysiological recordings [26,27]. The main challenge lies in the high temperature needed for carbon synthesis, incompatible with the use of polymer substrates [28]. Additionally, most of the carbon synthesis requires the use of a catalyst, such as nickel [29], iron [30], cobalt [31], and copper [32], which is potentially cytotoxic if not completely removed. Thanks to a pattern transfer technology, the integration of glassy carbon (GC) microelectrodes into flexible devices with metal interconnects has been achieved [33,34,35,36]. The GC microstructures are obtained from the pyrolysis of a pre-patterned polymeric precursor, such as SU-8, in an inert controlled atmosphere, avoiding risks of cytotoxicity and contamination and not requiring post-patterning.

GC MEAs, with GC microelectrodes and metal interconnections (*hybrid* MEAs), have shown highly sensitive neurotransmitter detection of DA and 5-HT [35] with good electrochemical fouling resistance [35] and high quality acute single-unit recordings [36,37]. *Hybrid* MEAs on thin flexible SU-8 substrates have also been demonstrated to reduce tissue damage and inflammation compared to stiff silicon MEAs, preserving a healthy neural tissue interface that can facilitate long-term sensing measurements [38].

The main concern faced with hybrid MEA fabrication is the adhesion between metal traces and GC electrodes, which is prone to failure upon prolonged electrical and mechanical stimulation. Using GC as a homogenous material for both electrodes and interconnects, without the need of adhesion layers, eliminates this concern. The first devices with GC electrodes and GC interconnects integrated on polyimide substrate have been fabricated by the Dr. Kassegne group and have shown outstanding electrochemical stability, demonstrating the capability of delivering charge-balance current pulsing over 3.5 billion cycles in an extended accelerated aging process lasting more than 1000 h, with no failure observed [21]. While this result holds promise for achieving stable neural interfaces, these devices require miniaturization [5] in order to reduce implantation injury and the inflammatory response for chronic electrochemical sensing.

Here, we show our progress in the fabrication of *all* GC-MEAs on thin flexible substrate while reducing dimensions to promote seamless tissue integration and long-lasting electrochemical recordings. We worked in parallel in the development of two microfabrication methods: (1) a double pattern-transfer photolithographic process, involving transfer-bonding on temporary polymeric support, similar to what was previously reported [21] and (2) a double-etching process, which uses a 2 µm-thick low stress low pressure chemical vapor deposition (LPCVD) nitride (Si_3_N_4_) coating of the Si wafer as the bottom insulator layer for the MEAs, bypassing the need for pattern-transfer and demonstrating a novel technique with potential advantages. This second process requires CF_4_ reactive ion etching of the Si_3_N_4_ to define the bottom shape of the device, after pyrolysis and patterning of the top MEAs insulation layer, and a purely chemical xenon difluoride (XeF_2_) etching of the exposed Si to release the Si_3_N_4_ insulated MEA from the Si wafer, taking advantage of the 200:1 Si versus Si_3_N_4_ selectivity [39,40].

We validated the feasibility of the proposed fabrication methods, by demonstrating the practical conductivity of 3 µm-wide 2 µm-thick GC traces and confirming the microelectrode functionality and discussed their respective strengths and limitations. Furthermore, we verified the ability of the GC microelectrodes to detect 5-HT using a FSCV N-shaped “modified Jackson waveform” (0.2 to 1.3 to −0.1 to 0.2 V) at a high scan rate (1000 V/s).

By sharing our progress in *all* GC-MEAs fabrication, we not only highlight the feasibility and potential of these methods but also encourage collaborative efforts and discussions within the community, fostering innovation and accelerating the development of novel neurotechnology applications. By sharing these results and insights, we hope to propel the development of GC-based MEAs for the next generation of neural interface devices.

## 2. Materials and Methods

### 2.1. Double Pattern-Transfer Microfabrication

Steps 1 and 2. A 4-in Si wafer with a 1 µm thick SiO_2_ layer (University Wafer Inc., Boston, MA, USA) was first cleaned with acetone, isopropanol, and deionized water (DI) sequentially. The wafer was then dried with a N_2_ spray gun, heated on a hot plate at 200 °C for 5 min, and treated with O_2_ plasma using a reactive ion etcher (RIE, MICRO-RIE 800, Technics Inc., Anaheim, CA, USA) for 90 s at 300 m Torr pressure and 150 W power. The cleaned wafer was spin-coated with SU-8 3010 (Kayaku Advanced Materials, Westborough, MA, USA) at 2000 rpm for 1 min and soft baked at 65 °C for 5 min and 95 °C for 5 min. Then, the wafer was exposed using a custom-made photomask and a MA/BA6 Mask/Bond Aligner (Süss MicroTec, Garching, Germany) with a dose of 350 mJ/cm^2^. After exposure, the wafer was first post-baked at 65 °C for 1 min and 95 °C for 3 min, then developed using SU-8 developer (Kayaku Advanced Materials, Westborough, MA, USA) for 1 min and cleaned with isopropanol and DI water. The patterned SU-8 was subsequently hard baked at 200 °C, 180 °C, and 150 °C for 5 min each and allowed to cool down below 65 °C. Pyrolysis of the negative SU-8 resist was performed in a high-temperature split tube furnace (STF 1200 Tube Furnace, Across International, Livingston, NJ, USA). The samples were heated to 900 °C with a temperature ramp-up at a rate of 3 °C/min, then maintained at 900 °C under 15 standard cubic centimeters per minute (sccm) N_2_ (Airgas, Pittsburgh, PA, USA) at 0.8 Torr for 60 min. The samples were then slowly cooled to room temperature. Step 3. After the pyrolysis, the wafer was cleaned with acetone, isopropanol, and DI water sequentially and treated with O_2_ plasma with RIE for 30 s at a pressure of 300 mTorr and 120 W power. The cleaned wafer was then spin-coated with SU-8 5 (Kayaku Advanced Materials, Westborough, MA, USA) at 3000 rpm for 1 min and then soft based at 65 °C for 3 min and 95 °C for 5 min. This SU-8 layer was patterned, using a dose of 300 mJ/cm^2^, to define the insulation layer. After a post-bake at 65 °C for 1 min and 95 °C for 3 min, the wafer was developed using the SU-8 developer. Finally, the patterned wafer was cleaned with isopropanol and DI water, hard baked at 200 °C, 180 °C, and 150 °C for 5 min each, and allowed to cool down below 65 °C. Step 4. After the hard baking, the wafer was treated with O_2_ plasma with RIE for 60 s at a pressure of 300 mTorr and 150 W power to improve the adhesion of the polydimethylsiloxane (PDMS, SYLGARD™ 184, Dow Corning, Midland, MI, USA) used as transfer substrate. Immediately after, the wafer was spin-coated with PDMS at 300 RPM for 20 s. PDMS was baked for 1 h at 80 degrees C and then allowed to cool down and stabilized overnight. Step 5 and 6. The 1st and 2nd layers of the MEAs were then transferred on the PDMS temporary transfer substrate using a wet chemical buffered oxide etching (BOE 7:1, Transene Co., Inc., Danvers, MA, USA) of SiO_2_ to release the PDMS from the Si substrate. Once the PDMS was completely released with the transferred MEAs, it was removed from the BOE bath and abundantly cleaned with DI water. Then, the standalone PDMS layer was flipped with the GC traces and interconnections of the MEAs facing up and bind to a Si wafer (temporary rigid support) to facilitate the next step, i.e., the insulation of the GC interconnections. The Si wafer was previously treated with O_2_ plasma with RIE for 90 s at a pressure of 300 mTorr and 150 W to facilitate the binding. Step 7. The temporary support was then spin-coated with SU-8 5 at 3000 rpm for 1 min and soft baked at 65 °C for 5 min and 95 °C for 5 min. Then, the wafer was exposed using a custom-made photomask and a MA/BA6 Mask/Bond Aligner with a dose of 350 mJ/cm^2^. After exposure, the wafer was first post-baked at 65 °C for 1 min and 95 °C for 3 min, then developed using a SU-8 developer (Kayaku Advanced Materials, Westborough, MA, USA) for 1 min, and then cleaned with isopropanol and DI water. The patterned SU-8 was subsequently hard baked at 200 °C, 180 °C, and 150 °C for 5 min each and allowed to cool down below 65 °C. Step 8. Finally, the insulated MEAs were gently pilled off from the PDMS.

### 2.2. Double Dry-Etching Microfabrication

Steps 1, 2, and 3: A 4-in Si wafer with a 2 µm-thick low stress LPCVD Si_3_N_4_ layer (University Wafer Inc., Boston, MA, USA) was first cleaned with acetone, isopropanol, and deionized water (DI) sequentially. The wafer was then dried with a N_2_ spray gun, heated on a hot plate at 200 °C for 5 min, and treated with O_2_ plasma using a reactive ion etcher (RIE, MICRO-RIE 800, Technics Inc., Anaheim, CA, USA) for 90 s at 300 mTorr pressure and 150 W power. These 3 steps are the same of the step 1–3 of the double pattern-transfer microfabrication. Briefly, the SU-8 precursor was patterned on the Si_3_N_4_-coated Si wafer followed by pyrolysis to obtain GC electrodes and interconnections and the SU-8 insulation layer was lithography patterned to define the device layout. Step 4. After the hard baking of the SU-8, the devices were protected with a sacrificial hard mask. First, the wafer was treated with O_2_ plasma with RIE for 60 s at a pressure of 300 mTorr and 100 W power and then spin-coated with a S1318 positive photoresist (MICROPOSIT™ S1800^®^ G2 Series Photoresists, Kayaku Advanced Materials, Westborough, MA, USA) at 800 rpm for 1 min and baked at 115 °C for 1 min. After soft baking, the wafer was exposed and patterned with a dose of 500 mJ/cm^2^, then developed using MF-321 developer (MICROPOSIT™, Kayaku Advanced Materials, Westborough, MA, USA), cleaned with water, rinsed, and dried by N_2_ gas flow. Step 5. Then the wafer was exposed to CF_4_ reactive ion etching (RIE, 220 mTorr pressure and 200 W power) to etch the 2 µm Si_3_N_4_ layer, where not protected, leaving the Si exposed. Step 6. To release the Si_3_N_4_-insulated MEA from the Si wafer, the exposed Si was etched using a purely chemical xenon difluoride (XeF_2_) etching with pressure P(XeF_2_) of 3.5 mT at room temperature, using a xenon difluoride etching tool (Xetch X.3.B, Xactix Inc., River Park Commons, PA, USA). Step 7. After the insulated MEAs were released, the sacrificial protective layer was easily removed with acetone.

### 2.3. Morphological and Chemical Characterization

Scanning electron microscope (SEM) imaging and elemental analysis of surfaces in field-emission electron microscopy were performed using energy dispersive spectroscopy (EDS) to identify and quantify all present elements using a HITACHI S-4800 field-emission electron microscope with a Bruker (Xflash 6160) EDS attachment (HITACHI Global, Irvine, CA, USA). High resolution optical imaging was performed using a VK-X150 3D scanning confocal microscope (Keyence America, Itasca, IL, USA).

### 2.4. Electrochemical Characterization

To verify the electrode functionality, electrochemical impedance spectroscopy (EIS) and cyclic voltammetry (CV) were performed in 1 × phosphate-buffered saline (PBS, Sigma Aldrich, St. Louis, MO, USA) in a three-electrode electrochemical cell set-up with a platinum counter electrode and an Ag/AgCl wire reference electrode, using a potentiostat/galvanostat (Autolab, Metrohm, Riverview, FL, USA). EIS was performed by superimposing a sine wave (10 mV RMS amplitude) onto the open circuit potential while varying the frequency from 1 to 10^5^ Hz. During the CV tests, the working electrode potential was swept between 1.3 and −0.6 V vs. Ag/AgCl at a scan rate of 100 mV/s.

Additionally, the sheet resistance of the GC traces (3 µm wide and 5 mm long) was measured on a Si substrate in comparison with gold traces of the same width and length. To do so, 3 µm wide and 5 mm long traces (Au and GC) were fabricated on a silicon substrate together with 3 × 3 mm pads to provide reliable connection points for the measurement probes. Then, the resistance was measured using EIS in a two-terminal setup (in the air) and the sheet resistance was calculated knowing the thickness, length, and width of the traces.

### 2.5. Fast Scan Cyclic Voltammetry Sensing

Fast scan cyclic voltammetry (FSCV) measurements of 5-HT were collected using a FSCV Wave Neuro potentiostat connected to a Flow Cell System (Pine Research, Durham, NC, USA) to validate the GC microelectrode sensitivity and analyzed using HDCV software (University of North Carolina at Chapel Hill, Chapel Hill, NC, USA). The electrode was scanned using a modified N-shaped waveform (0.2 to 1.3 to −0.1 to 0.2 V) at 10 Hz and 1000 V/s scan rate. 5-HT was identified by inspection of the background-subtracted cyclic voltammograms. Electrodes were calibrated using 0.1–1.5 μM 5-HT concentrations dissolved in 1× PBS. The different concentrations were diluted starting from freshly prepared 1 mM 5-HT solutions. Calibration was performed in a flow cell Flow Cell System (Pine Research, Durham, NC, USA). Flow through the system at 60 mL/h was driven with a syringe pump.

## 3. Results and Discussions

### 3.1. Patterning and Carbonization of GC Electrodes and Interconnections

**Figure 1:**
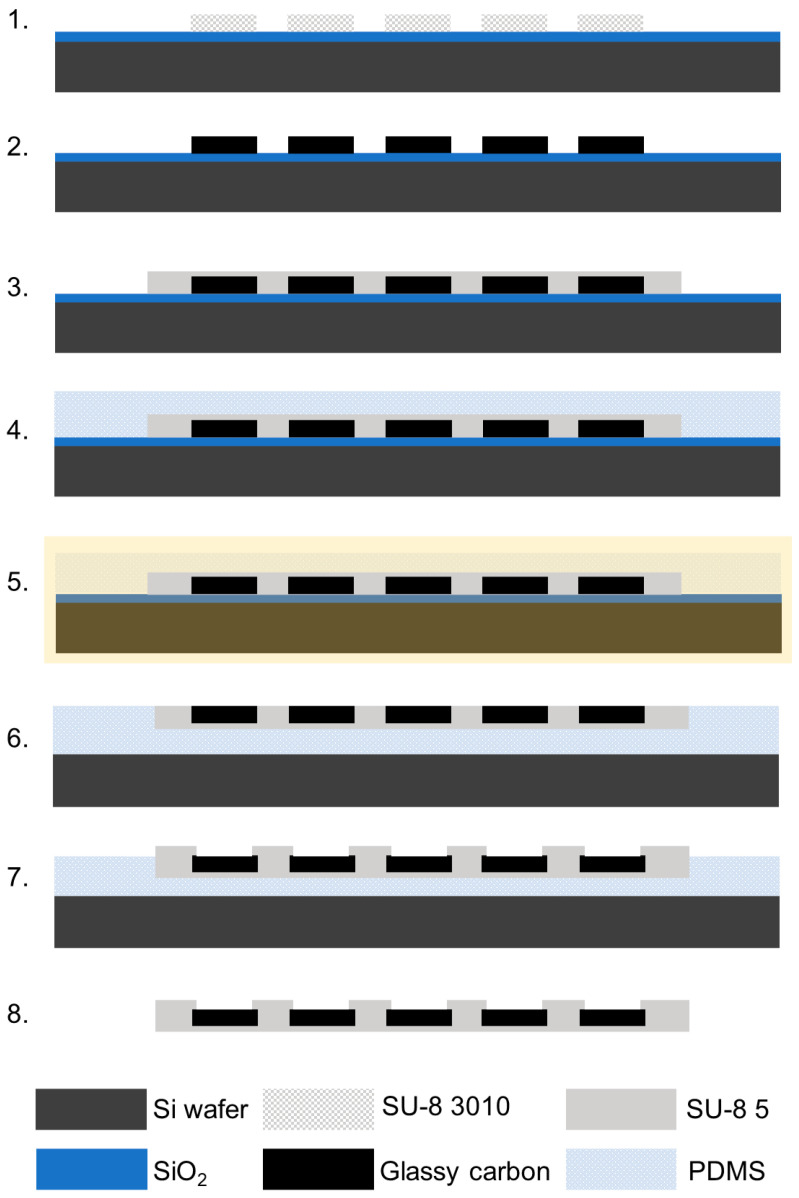
Schematic for double pattern-transfer microfabrication of *all* GC MEAs: (1.) SU-8 3010 spin-coating and patterning of electrodes on SiO_2_ wafer; (2.) pyrolysis; (3.) SU-8 insulation layer spin-coating on top of the GC electrodes and UV exposure to pattern the insulation layer; (4.) deposition of the temporary transfer substrate (Polydimethylsiloxane PDMS); (5.) wet chemical-buffered oxide etching of SiO_2_ to release PDMS from Si substrate, transferring the MEAs on PDMS; (6.) binding of the released PDMS transfer layer with the GC traces and interconnections of the MEAs facing up to a Si wafer (temporary rigid support); (7.) SU-8 top insulation layer spin-coating and patterning of the probe outline; and finally, (8.) the insulated MEAs were gently pilled off from the PDMS.

**Figure 2:**
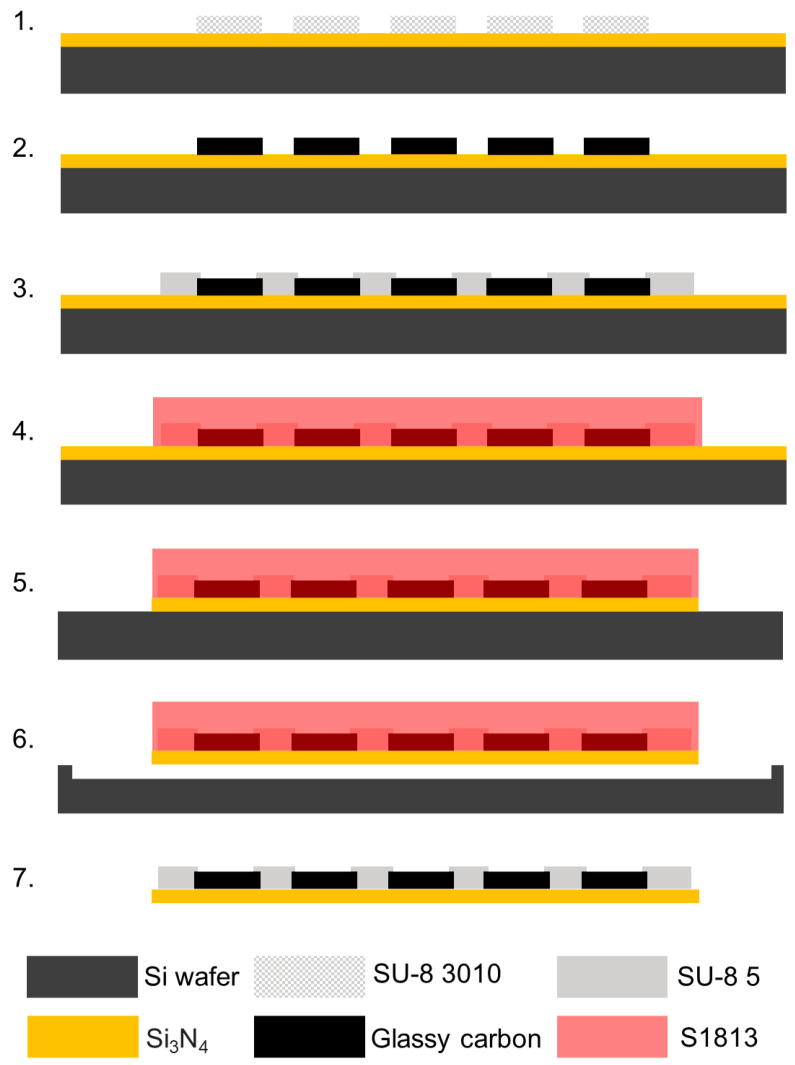
Schematic for double dry-etching microfabrication of *all* GC MEAs: (1.) SU-8 3010 spin-coating and patterning of electrodes on SiO_2_ wafer; (2.) pyrolysis; (3.) SU-8 insulation layer spin-coating on top of the GC electrodes and UV exposure to pattern the insulation layer; (4.) spin-coating and photolithographic patterning of positive photoresist S1813 (protective sacrificial layer); (5.) CF_4_ reactive ion etching (RIE) of the not protected Si_3_N_4_; (6.) pure chemical isotropic XeF_2_ etching of the exposed Si substrate to release the devices; and (7.) after the insulated MEAs were released, the sacrificial protective layer was easily removed with acetone.

*All* GC-MEAs were successfully developed following the two fabrication processes schematically described in Figure 1 and Figure 2. The first step, common to both the methods, is the patterning and carbonization of SU-8 to obtain miniaturized GC features. Different MEA layouts were incorporated into the same photomasks to test different trace spacing (10 to 20 µm), microelectrode center-to-center distances (100 to 250 µm), microelectrode shapes (30 to 40 µm diameter circular or 55 × 25 µm oval), microelectrode numbers (5 to 8), and shank widths (90 to 140 µm). Supplementary Figure S1 reports an example of batch-fabricated GC microelectrodes and interconnections.

**Figure 3:**
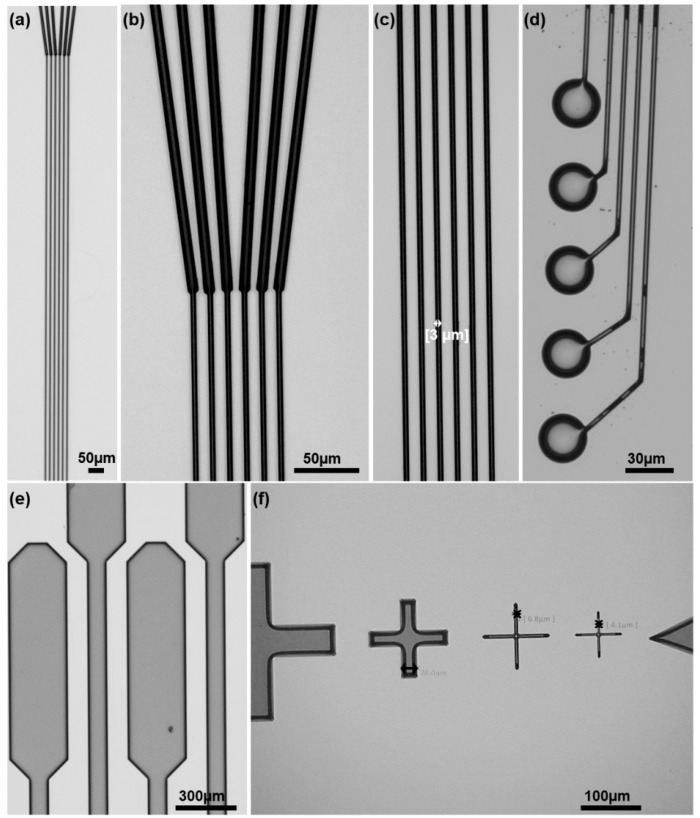
Glassy carbon (GC) features on silicon substrate: (**a**–**c**) traces and interconnects, (**d**) microelectrodes, (**e**) connection pads, and (**f**) aligner marks.

Figure 3 shows the results from the patterning and carbonization of 3 µm-wide GC traces (a–c), 30 µm diameter microelectrodes (d), 300 µm pads (e), and different size aligner markers (f), with features down to 3 µm, for high alignment accuracy. The sheet resistance of the 3 µm wide and 5 mm long GC traces was measured on Si substrate, in comparison with gold traces of the same width and length. The value obtained is 1.21 ± 0.26 Ω/sq, which is the same order of magnitude as gold traces of the same dimension (0.22 ± 0.01 Ω/sq). Thus, we do not expect signal attenuation due to the large serial resistance of the GC traces. These values have been obtained for GC traces approximately 2 µm thick. We observed that during the pyrolysis, the hard-baked SU-8 precursor experienced about 70% shrinkage. This factor should be considered for the choice of SU-8 viscosity and spinning rate during the photolithography. Controlling these parameters effectively is crucial for achieving the desired thickness and uniformity of the SU-8 layer, which ultimately determines the dimensions and properties of the fabricated GC traces.

#### 3.1.1. Double Pattern-Transfer Microfabrication

**Figure 4:**
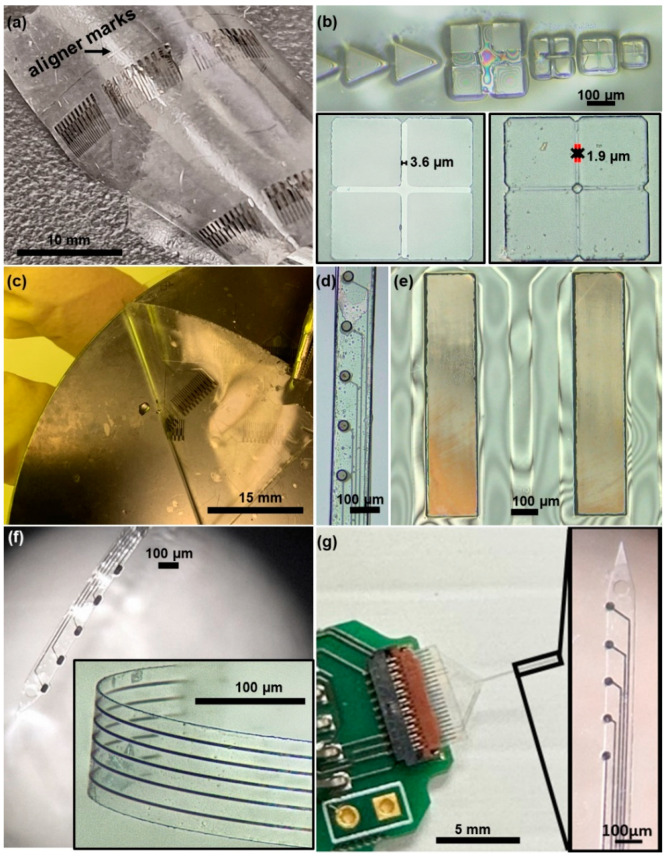
Double pattern transfer fabrication: (**a**) standalone PDMS layer (300 µm thick) containing the transferred *all* GC-MEAs, after being released from the SiO_2_ wafer using a chemical buffered oxide etching, (**b**) zoom on the aligner marks on the standalone PDMS (GC first layer and SU-8 second layer), (**c**) PDMS layer transfer-bonded to a rigid silicon substrate to offer temporary support, (**d**,**e**) magnification of the microelectrodes and connecting pads post-insulation, (**f**) final insulated *all* GC-MEA (140 µm wide and ~8 µm thick, oval GC electrodes) during the peeling off from the PDMS (zoom of the folding in inset), and (**g**) finally insulated *all* GC-MEA connected to the custom-made printed circuit board (PCB) using a zero-insertion force (ZIF) connector to be interfaced with the potentiostat for characterization and sensing. The corresponding magnification of the shank in the inset (120 µm wide and ~8 µm thick, 30 µm diameter circular GC electrodes).

Figure 4a and Figure S2 report standalone PDMS layers (250–300 µm-thick) containing the transferred *all* GC-MEAs, after being released from the SiO_2_ wafer using chemical-buffered oxide etching. This step involves separating the PDMS layer, along with *all* GC-MEAs, from the SiO_2_ wafer, thus exposing the side of the GC electrode, traces, and interconnections that still need to be insulated. The precision of the aligner marks on PDMS, reported in Figure 4b, highlights the success of one of the most critical steps, i.e., the transferring of the first and second layers of the MEAs onto the PDMS temporary transfer substrate. Both SU-8 and GC have shown a good adhesion to PDMS, after the oxygen plasma treatment, achieving proper bonding. This adhesion ensures that the layers (SU-8 and GC) firmly attach to the PDMS substrate, which is critical for maintaining structural integrity and functionality throughout the fabrication process. PDMS is highly elastic and flexible, making it ideal for replicating intricate patterns with high fidelity and conforming to various shapes, thus facilitating this delicate transferring process [41]. Additionally, PDMS presents good chemical inertness; it does not react with most chemicals [42] and it was able to sustain BOE oxide etching. Subsequently, the standalone PDMS layer, with the GC traces and interconnections of the MEAs facing up, was transfer-bonded to a rigid silicon substrate to offer temporary support, as illustrated in Figure 4c. The use of a rigid silicon substrate facilitates precise alignment for the next step, which involves insulating the GC interconnections while ensuring well-aligned openings on the pads and electrodes, crucial to ensure reliable electrical connections and electrochemical readings. Figure 4d,e shows the magnification of the microelectrodes and connecting pads post-insulation, highlighting the aligned openings in the final insulation layer. PDMS also presents low free surface energy, which favors the peel-off of the finalized MEAs from the PDMS [42,43]. Furthermore, it is biocompatible and non-toxic, minimizing any concern of possible contaminations. Figure 4f shows a finally insulated *all* GC-MEA while peeling off from the PDMS. This MEA is composed of a singular shank (140 µm wide and ~8 µm thick) with five oval GC electrodes.

Figure 4g shows the finally insulated *all* GC-MEA, with the corresponding magnification of the shank, connected to the custom-made printed circuit board (PCB) using a zero-insertion force (ZIF) connector, to be interfaced with the potentiostat for characterization and sensing. At the tip of the shank, an anchor hole was patterned in the SU-8 insulation layers to facilitate the insertion of a 50 µm tungsten shuttle that will enable the handling and penetration of the probes into the brain, as previously reported. This device is composed of a singular shank (120 µm wide and ~8 µm thick) with five circular GC electrodes 30 µm in diameter. The total length of the shank is 5 mm to easily target the deep brain region of the mice [38].

These results demonstrate the feasibility of the double pattern-transfer photolithographic process for the fabrication of *all* GC-MEAs on flexible SU-8 substrate with miniaturized features. However, limitations emerged due to the complexity of this process, particularly regarding further miniaturization and scalability. The PDMS transfer method was effective in transferring *all* GC-MEAs but handling large PDMS sheets posed difficulties during batch fabrication, limiting the fabrication to small areas. Indeed, the positioning of a 300 µm-thick standalone PDMS of a larger area on the silicon resulted in micro-folding and shifting, which significantly impact the alignment of devices, limiting the number of devices that can be processed at once. Currently, only small sections of PDMS can be successfully used, as observed in Figure 4a. Thicker PDMS layers have shown poor adherence and bonding to the rigid Si substrate. However, optimizing the deposition process and tailoring PDMS properties are crucial steps to further enhance its adherence and bonding to the rigid silicon substrate. Adjusting parameters such as the curing temperature, time, or surface treatments for both PDMS and the silicon substrate could significantly improve their compatibility [44,45]. Due to the advantageous properties of the PDMS, exploring a mold-based approach for thicker and more stable PDMS transfer seems like a viable solution to consider. Using a higher crosslinker concentration, adding modifying agent or reinforcement fillers can increase the PDMS stiffness [45,46]. Employing a more rigid and flat-molded PDMS of optimized thickness might eliminate the need for additional rigid support during the transfer process. Thorough testing and optimization will be essential to ensure that the modified PDMS maintains its advantageous properties while addressing the challenges encountered during fabrication. Moreover, considering alternative temporary transfer materials could be beneficial. These materials should strike a balance between various mechanical and chemical properties based on the process needs, such as conforming and bonding to SU-8 of different shapes, chemical inertness, and easy removal. These discussed approaches—fine-tuning PDMS properties, exploring mold-based transfers, and investigating alternative transfer materials—can potentially address the challenges faced in handling larger PDMS sheets and improve the scalability and efficiency of batch fabrication.

#### 3.1.2. Double Dry-Etching Microfabrication

Here, an alternative microfabrication method that does not require a double pattern-transfer technique to develop *all* GC-MEAs with miniaturized features was also developed and tested. In this process, referred to as double dry-etching microfabrication, the fabrication of the GC and insulation layer was repeated on 2 µm-thick low-stress LPCVD nitride Si_3_N_4_ wafers. Briefly, the SU-8 precursor was patterned on the Si_3_N_4_ wafer followed by pyrolysis to obtain GC electrodes and interconnections with trace width and thickness optimized to ensure practical conductivity. Second, the SU-8 insulation of the device was patterned and hard-backed. Subsequently, the MEAs were protected with a sacrificial hard mask to preserve the insulation and GC contact in the subsequent CF_4_ RIE etching process of the non-protected Si_3_N_4_ covering the wafer. This process was able to selectively etch away the Si_3_N_4_ while preserving the insulated GC-MEA features. The Si_3_N_4_ etching rate was 108.28 ± 7.9 nm/min, taking 20 min to completely expose all the underneath Si substrate. At this point, the GC traces, microelectrodes, and interconnections were insulated on the bottom from Si_3_N_4_ and on the top by SU-8 and they were required to be released by the Si substrate.

Si_3_N_4_ has been extensively used as an insulator substrate for microfabricated multielectrode arrays and it has been shown to offer several advantages such as biocompatibility, good electrical insulation, mechanical robustness, and chemical stability [47,48,49]. Despite Si_3_N_4_ having an elastic modulus of hundreds of GPa [50,51], significantly higher compared to polymeric substrates, such as SU-8 [52] and polyimide [53], the fabrication of ultrathin semiconductor membranes allows silicon-based electronics to achieve extraordinary flexibility [54,55]. Moreover, ultrathin silicon layers transferred onto flexible polymers can potentially adapt to the flexibility offered by polymers [56,57].

**Figure 5:**
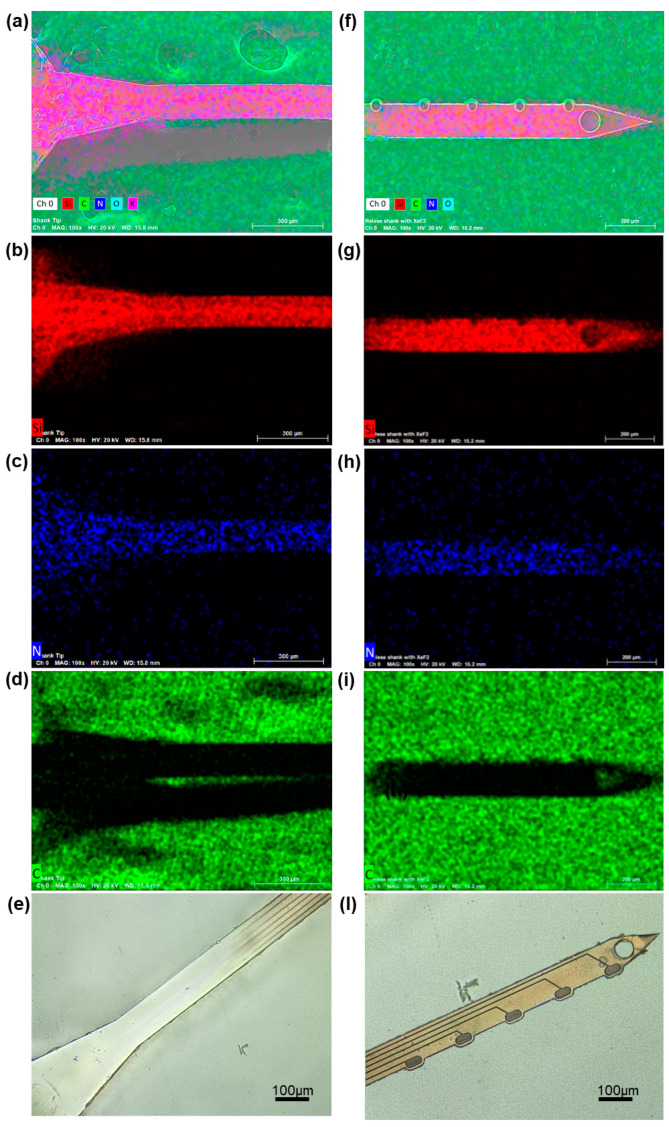
Double dry-etching microfabrication. SEM images and elemental analysis of the surface of *all* GC-MEA release using isotropic XeF_2_ etching (side insulated with Si_3_N_4_): (**a**,**f**) composition of carbon, silicon, nitrogen, and oxygen; (**b**,**g**) silicon in red; (**c**,**h**) nitrogen in blue; and (**d**,**i**) carbon in green. (**e**,**l**) Optical picture of the final insulated *all* GC-MEA: magnification of different areas along the shank (120 µm wide and ~6 µm thick, oval GC electrodes).

At this stage, a purely chemical XeF_2_ etching was used to release *all* GC-MEAs from the Si substrate, involving the use of XeF_2_ gas to selectively remove Si in a uniform manner. XeF_2_ gas selectively reacts with Si atoms on the surface, without the need for a liquid phase, leading to the removal of silicon atoms in the form of volatile silicon fluoride (SiF_4_) [39]. The gas adsorbs uniformly onto the silicon surface, initiating isotropic removal in all directions, enabling precise depth etching without damaging surrounding materials. XeF_2_’s selectivity ensures minimal reactivity towards materials like SiO_2_, SiC, Si_3_N_4_, and photoresist, preserving their integrity during the silicon etching process [40,58]. In this work, using a pressure of 3.5 mTorr at room temperature, the Si etching rate was 1.254 ± 0.166µm/min. The process demonstrated high selectivity, allowing precise etching of the Si substrate while maintaining the integrity of Si_3_N_4_ layers, as observed performing EDS analysis (Table 1, Figure 5). Previous studies under similar conditions did not observe etching of SiO_2_ or Si_3_N_4_, indicating an extremely large selectivity between silicon and its compounds [39,40]. Figure 5 reports the SEM images with elemental analysis of the surface of *all* GC-MEAs released using isotropic XeF_2_ etching (side insulated with Si_3_N_4_). The elemental analyses conducted using energy dispersive X-ray spectroscopy (EDS) confirmed a consistent and uniform presence of Si and N along the shank of the MEAs and the presence of carbon in the areas where the focus of the analysis was on the carbon tape of the stub (where the MEAs are positioned for imaging).

Overall, our results suggest that the double dry-etching microfabrication offers a promising method for producing *all* GC-MEAs on flexible substrates with miniaturized features. Its key advantage lies in circumventing the need for double pattern transfer, streamlining the fabrication process significantly and reducing potential errors associated with transferring and aligning micrometer feature patterns. This approach will facilitate further miniaturization of *all* GC neural probes. With further optimization and refinement, this technique could lead to more reliable and scalable manufacturing of *all* GC-MEAs and carbon-based neural interface technologies.

However, there are some challenges that need to be discussed associated with maintaining process control [59]. Maintaining high selectivity between silicon and silicon nitride during XeF_2_ etching is crucial for ensuring the reliability and functionality of devices. Therefore, different factors that can influence the etch rate and selectivity should be carefully considered during the design and optimization of the etching process, including the temperature, pressure, exposure time to XeF_2_ gas, addition of gases, such as hydrogen, and process variability [59]. Additionally, controlling the level of XeF_2_ supplied to the chamber is crucial in the XeF_2_ etching processes. The amount of XeF_2_ introduced significantly influences the etch rate and is a major source of variability. The amount of solid XeF_2_ within the bubbler determines the initial supply, which slowly decreases over time as the etching process continues. Running etch processes in rapid succession can deplete the available XeF_2_ more quickly. The etching rate is also affected by the quantity of silicon in the chamber. More silicon means a faster consumption rate, impacting the etch rate [60]. To achieve a reasonable and consistent etch rate, managing these factors becomes critical. Limiting the batch fabrication to smaller pieces of wafer at a time could mitigate this issue but restrict the scalability.

Transitioning to industrial-grade etching equipment might offer a potential solution to some challenges associated with process control, enabling more precise regulation of parameters like gas flow rates, pressure, and substrate handling. These systems are designed to manage larger quantities of substrates while maintaining more stable and controlled etching conditions, potentially addressing the challenges faced with fluctuating etch rates due to XeF_2_ supply variability [61]. However, despite the potential benefits, it is essential to consider the associated safety, environmental impact, cost, and availability of industrial equipment in making such a transition. Striking a balance between the benefits and limitations of XeF_2_ etching requires comprehensive strategies that consider process optimization, safety precautions, cost-effectiveness, and strict adherence to safety protocols. Technological and research progress seeks to address these limitations while optimizing the effectiveness of XeF_2_ etching in microfabrication applications.

### 3.2. Electrochemical Characterization and Sensing

**Figure 6:**
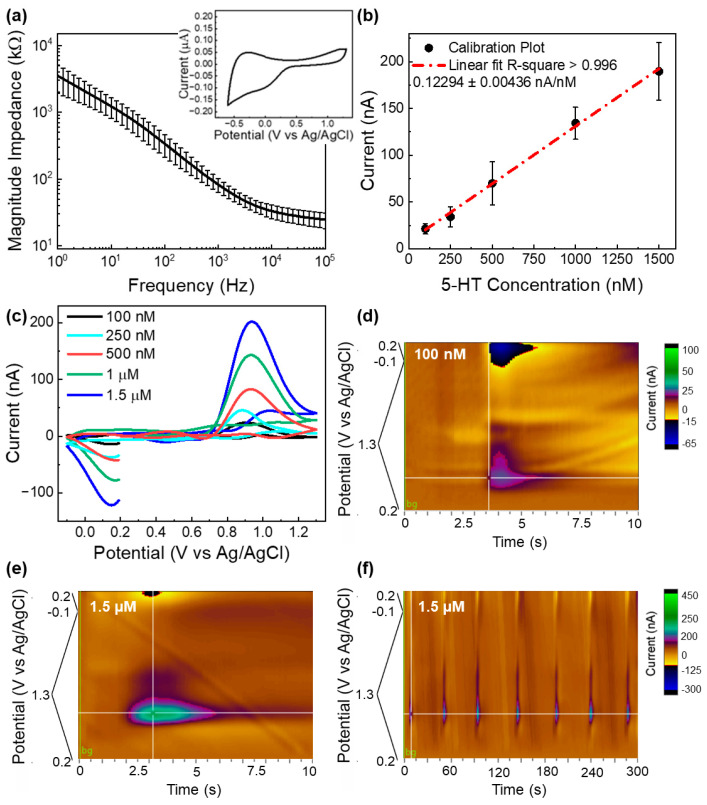
Electrochemical and FSCV characterization. (**a**) Electrochemical impedance spectra of the magnitude impedance of GC microelectrodes (mean and SD, n = 6, (4 for pattern transfer and 2 for double etching methods). In inset: representative example of a CV plot of a GC electrode; (**b**) in vitro FSCV calibration conducted at GC microelectrodes in 1× PBS in the 5-HT concentration range of 0.1–1.5 μM (peak current vs. 5-HT concentration, mean and SD, n = 5, (three for pattern transfer and two for double etching methods); (**c**) representative background subtracted FSCVs corresponding to the considered concentration range, demonstrating clear oxidation peaks at 0.9 V and reduction peaks at 0.15 V vs Ag/AgCl; (**d**) representative color plot corresponding to the detection of 100 nM bolus injection of 5-HT, (**e**) representative color plot corresponding to the detection of 1.5 μM bolus injection, and (**f**) multiple consecutive repetitions (every 45 s) of 1.5 μM 5-HT bolus injections over a 5-min FSCV collection.

EIS measurements (Figure 6a) resulted in impedance values of 30µm diameter GC microelectrodes (82.622 ± 19.218 @ 1 kHz), comparable with the GC microelectrodes of *hybrid* MEAs of a similar size [36]. The cyclic voltammogram revealed a wide electrochemical window extending up to 1.3 V without inducing hydrolysis reactions. This broad potential range should allow FSCV detection without compromising the background stability. The measurements have been performed using microelectrodes from different MEAs fabricated using both methods.

Finally, GC microelectrodes of *all* GC-MEAs were subjected to FSCV measurement for the detection of 5-HT in the 0.1–1.5 μM concentration range, using an N-shaped “modified” Jackson waveform (0.2 to 1.3 to −0.1 to 0.2 V) at a 1000 V/s high scan rate. This waveform should exclude the most likely interference from DA and other catecholamines because the scan rate has been shown to be too fast for DA to undergo the redox reaction using GC microelectrodes [35]. Such an N-shaped waveform has also been shown to be highly effective in reducing electrode fouling [35,62]. The GC microelectrodes exhibited high sensitivity to 5-HT (122.94 ± 4.36 nA/μM) in the concentration range of 0.1–1.5 μM, with a linear (r^2^ > 0.99, Figure 6b) response. Figure 6c shows the background subtracted CVs corresponding to the considered concentration range, demonstrating clear oxidation peaks at 0.9 V and reduction peaks at 0.15 V vs. Ag/AgCl. Figure 6d illustrates a representative color plot corresponding to the detection of a 100 nM bolus injection of 5-HT, underlining the possibility to clearly identify 5-HT reduction and oxidation peaks in the concentration range usually detectable in the mouse brain using FSCV [63,64]. Figure 6e,f report color plots corresponding to a response to a much higher 5-HT concentration of 1.5 μM. No background drift was observed during multiple repetitions of 1.5 μM 5-HT bolus injections over a 5-min FSCV collection in 1× PBS (Figure 6f), underlining good stability of the background current. These results collectively confirmed the GC-MEA functionality and capability for selective and stable electrochemical detection of 5-HT. The findings highlight the potential for further comprehensive testing, both in vitro and in vivo, to assess electrochemical and sensing stability for chronic implants. 

5-HT imbalance has been shown to be implicated in various neurological and psychiatric disorders, such as depression, anxiety, chronic pain, and impulse control disorders but the underlying mechanisms are still unclear. Thus, the potential of 5-HT measurement, in combination with electrophysiological recording, would enable a deeper understanding of the role of 5-HT in these conditions and consequently improve the efficacy of specific pharmacological treatments.

## 4. Conclusions

This work showcases the successful development of “all” GC-MEAs on thin flexible substrates aimed at enhancing tissue integration and ensuring long-lasting electrochemical recordings.

We pursued two parallel paths in fabrication techniques:

(1) A double pattern-transfer photolithographic process, involving transfer-bonding on temporary polymeric support, and (2) a double-etching process which uses the 2 µm-thick low stress LPCVD nitride coating of the Si wafer as bottom insulator layer for the MEAs, bypassing pattern transfer and demonstrating a novel technique with potential advantages;
(2) A novel double-etching process utilizing a 2 µm-thick low-stress LPCVD nitride coating as the bottom insulator layer, bypassing pattern transfer and offering potential advantages. This latter technique involves CF4 reactive ion etching of Si3N4 to define the bottom device shape, followed by a chemical XeF2 etching to release the Si3N4-insulated MEA from the Si wafer, taking advantage on high Si versus Si3N4 selectivity.

We presented step-by-step fabrication results and discussed the advantages and limitations of the two fabrication techniques. We demonstrated the practical conductivity of 3 µm-wide 2 µm-thick GC traces and validated the microelectrode functionality. Utilizing FSCV with an N-shaped “modified Jackson waveform” at a high scan rate (1000 V/s), we successfully detected 5-HT, confirming the sensing capability of the GC microelectrodes.

Presenting our results in *all* GC-MEAs fabrication not only highlights the feasibility and potential of these methods but also encourages collaborative efforts and discussions within the community, fostering innovation and accelerating the development of novel neurotechnology applications. The discussions of the respective strengths and limitations of the two fabrication methods will undoubtedly provide crucial insights for refining and developing the next generation of neurotechnology devices, leading to improved designs and functionalities that can further advance the field. Optimization of this new class of neural devices is urgently needed. By sharing our progress, we hope to encourage collaborative efforts and discussions within the community, accelerating their development and characterization.

## Figures and Tables

**Table 1:** EDS analysis with a relative percentage of chemical elements.

Element	Mass Normalized (%)	Abs Error (%)	Rel. Error (%)
Carbon	56.336 ± 3.927	8.222 ± 0.998	11.636 ± 0.216
Nitrogen	15.344 ± 3.974	3.108 ± 0.826	16.288 ± 2.126
Oxygen	11.340 ± 3.749	1.992 ± 0.356	14.546 ± 0.801
Silicon	16.400± 4.202	0.929 ± 0.324	4.382 ± 0.120

## Data Availability

The data presented in this study are available on request from the corresponding author (elisa@latech.edu).

## Supplementary Materials

The following supporting information can be downloaded at: https://www.mdpi.com/article/10.3390/mi15020277/s1, Figure S1: GC microelectrode and interconnection on a 4-inch wafer. Different MEA layouts were incorporated into the same photomasks to test different trace spacing (10 to 20 µm), microelectrode center-to-center distances (100 to 250 µm), microelectrode shape (30 to 40 µm diameter circular or 55 × 25 µm oval) and microelectrode numbers (5 to 8), shank width (90 to 140 µm). Figure S2: Double Pattern Transfer Fabrication: (a,b) optical images of two standalone PDMS layers (300 µm thick) containing the transferred all GC-MEAs, after being released from the SiO_2_ wafer using a chemical buffered oxide etching.
