# Supplementary material for: Batch Fabrication of Microelectrode Arrays with Glassy Carbon Microelectrodes and Interconnections for Neurochemical Sensing: Promises and Challenges
Source: Micromachines (Basel). 2024 Feb 15;15(2):277. doi: 10.3390/mi15020277 (PMC10892456; doi:10.3390/mi15020277)
Supplement: Supplementary file 1 [file micromachines-15-00277-s001.zip › micromachines-2856693-supplementary.pdf]

Supplementary Information: Batch fabrication of microelectrode arrays with glassy carbon microelectrodes and interconnections for neurochemical sensing: promises and challenges

Emma-Bernadette A. Faul [1], Austin M. Broussard [1], Daniel R. Rivera [1], May Yoon Pwint [2,3], Bingchen Wu [2,3], Qun Cao [2], Davis Bailey [4], X. Tracy Cui [2,3,5] and Elisa Castagnola [1,2,*]

[1] Department of Biomedical Engineering, Louisiana Tech University, Ruston, LA 71272, USA; eaf021@email.latech.edu (E.-B.A.F.); austinbrouss@gmail.com (A.M.B.); drr029@latech.edu (D.R.R.)
[2] Department of Bioengineering, University of Pittsburgh, Pittsburgh, PA 15260, USA; m.pwint@pitt.edu (M.Y.P.); biw15@pitt.edu (B.W.); qc3bs@virginia.edu (Q.C.); xic11@pitt.edu (X.T.C.)
[3] Center for Neural Basis of Cognition, University of Pittsburgh, Pittsburgh, PA 15261, USA
[4] Institute for Micromanufacturing, Louisiana Tech University, Ruston, LA 15213, USA; dbailey@latech.edu
[5] McGowan Institute of Regenerative Medicine, University of Pittsburgh, Pittsburgh, PA 15219-3110, USA
* Correspondence: elisa@latech.edu

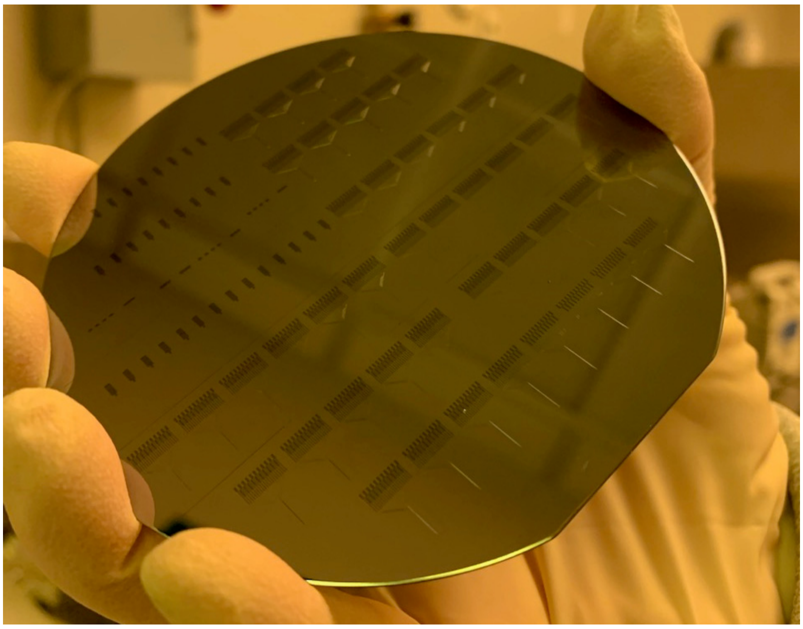

Figure S1. GC microelectrode and interconnection on a 4-inch wafer. Different MEA layouts were incorporated into the same photomasks to test different trace spacing (10 to 20 μm), microelectrode center-to-center distances (100 to 250 μm), microelectrode shape (30 to 40 μm diameter circular or 55 x 25 μm oval) and microelectrode numbers (5 to 8), shank width (90 to 140 μm).

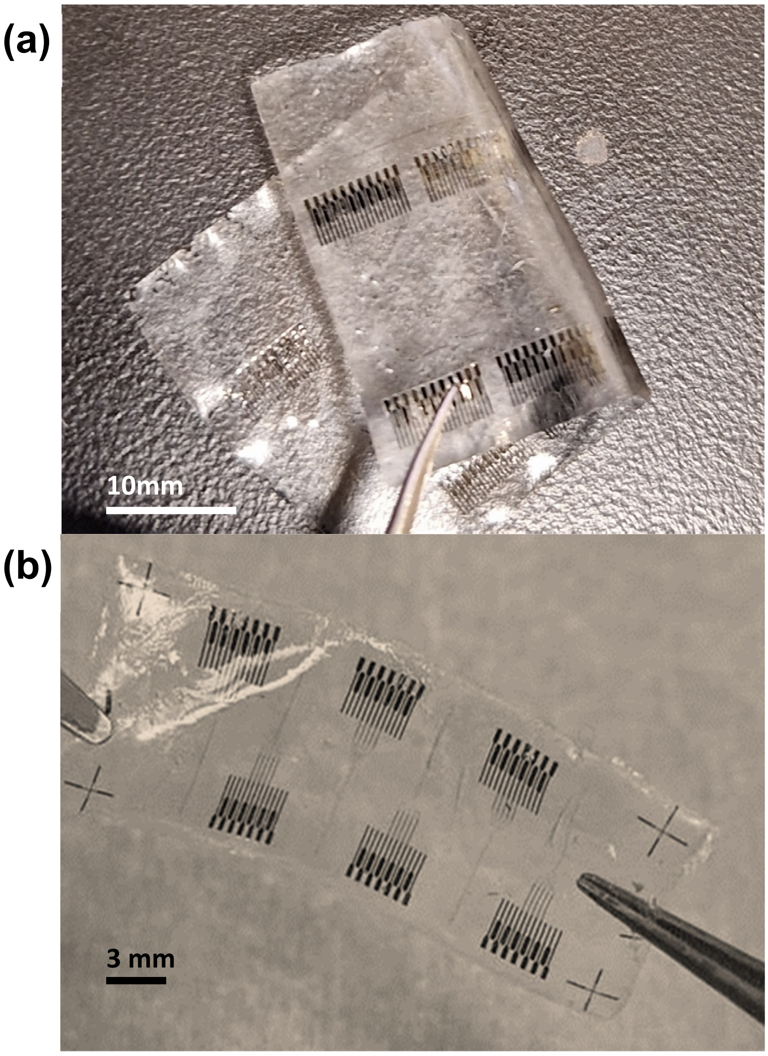

Figure S2. Double Pattern Transfer Fabrication: (a, b) optical images of two standalone PDMS layers (300 μm thick) containing the transferred all GC-MEAs, after being released from the SiO2 wafer using a chemical buffered oxide etching.
